# “Are Filipinos Aging Well?”: Determinants of Subjective Well-Being among Senior Citizens of the Community-Based ENGAGE Study

**DOI:** 10.3390/ijerph17207636

**Published:** 2020-10-20

**Authors:** Rogie Royce Carandang, Akira Shibanuma, Edward Asis, Dominga Carolina Chavez, Maria Teresa Tuliao, Masamine Jimba

**Affiliations:** 1 Department of Community and Global Health, Graduate School of Medicine, The University of Tokyo, Tokyo 113-0033, Japan; shibanuma@gmail.com (A.S.); mjimba@m.u-tokyo.ac.jp (M.J.); 2 Department of Global Studies, Faculty of Liberal Arts, Sophia University, Tokyo 102-8554, Japan; edward.asis2015@gmail.com; 3 Office for Senior Citizens Affairs-Muntinlupa, Muntinlupa City 1770, Philippines; domingacarolinac@yahoo.com; 4 City Health Office-Muntinlupa, Muntinlupa City 1770, Philippines; materesatuliao63@gmail.com

**Keywords:** cross-sectional study, gerontology, mental health, Philippines, psychosocial factors, subjective well-being

## Abstract

As people age, they are expected to experience adverse life conditions and major life events. These circumstances might have a significant impact on their subjective well-being. This study investigated the factors associated with subjective well-being among community-dwelling Filipino senior citizens. We conducted a cross-sectional study among 1021 senior citizens (68.5% women) aged 60 and above and identified the factors independently associated with their subjective well-being using multiple linear regression analysis. We also used hierarchical regression analysis for model prediction. In the hierarchical regression analysis, psychological resilience was found as the most powerful predictor of subjective well-being. Loneliness, however, was the only psychosocial factor not associated with it. Both men and women with positive self-rated health and had higher psychological resilience and perceived social support showed a higher level of subjective well-being. Women who were separated and received pension and men who were uneducated showed a lower level of subjective well-being. Psychological resilience, positive self-rated health, and perceived social support might be protective factors for low subjective well-being. To improve the subjective well-being of Filipino senior citizens, we should build psychological resilience and social support networks in the community.

## 1. Introduction

The study of subjective well-being seeks to understand individuals’ assessment of their lives. It refers not only to the absence of mental disorders but to the individual’s positive evaluation of their experience and psychological functioning [1]. Subjective well-being has two aspects. First, the “hedonic or experiential well-being” refers to the pursuit of happiness and pleasant life. It also refers to the extent to which people experience positive effects (e.g., calmness or happiness) and negative effects (e.g., worry, sadness, or anger) in their daily lives [2,3,4]. Secondly, “eudaimonic or evaluative well-being” refers to human development and a meaningful life. It comprises the cognitive evaluations that people make about their life satisfaction [5,6]. Despite the distinction, subjective well-being is considered a multidimensional construct [7,8,9], and such multidimensionality has led to a different and confusing research base.

Previous studies have tried to ascertain whether there are life-course effects on subjective well-being at the population level. Mixed results were obtained accounting for differences across the studies in terms of design, sampled population, and data analysis. Some studies showed that subjective well-being is stable or increasing well into old age [10,11]. For instance, according to a review of large-scale international studies of individuals aged 20–80+, subjective well-being showed no decline with age in most societies [10]. On the other hand, other studies showed that subjective well-being is U-shaped through the life cycle in Western countries with a minimum level of subjective well-being occurring around midlife [12,13]. They have also seen a hill-shaped relationship between mental disorders (e.g., depression and anxiety) and age [12]. Thus, middle-aged individuals were vulnerable to low subjective well-being and mental disorders. However, with increasing age, people are expected to experience accumulation of adverse life conditions (e.g., disease and disability) and major life events (e.g., loss of companionship) [14,15]. These circumstances might have a significant impact on their subjective well-being. One longitudinal study supported this hypothesis and showed that subjective well-being decreased only after the age of 70 [16]. Hence, the challenge lies within understanding the factors that may affect the subjective well-being of the aging population. 

The determinants of subjective well-being among senior citizens include socioeconomic status [17], psychological resources [18], social capital [19], and social relationships [20]. In a systematic review conducted among senior citizens in Europe, a lower socioeconomic status was associated with poorer subjective well-being [21]. Socially meaningful relations were positively associated with subjective well-being and quality of life among senior citizens in the United States [22,23]. Evidence suggests that living with a partner or being married can positively impact life satisfaction [24] and is associated with higher subjective well-being among older Europeans [25]. However, the differences in societies and gender have not been explored yet. This focus is essential, given that the aging experience may differ between societies [26] and the different role trajectories of men and women [27]. For instance, people in individualistic societies (e.g., United States, United Kingdom, the Netherlands, and Germany) tend to focus on their own living conditions. In contrast, people in collectivistic societies (e.g., Japan, South Korea, China, and Thailand) tend to consider their family’s well-being when they evaluate their subjective well-being [28,29]. Women also typically live longer than men, and older women tend to become widowed or spousal caregivers to men. This situation may have affected women’s subjective well-being [15]. As most of the relevant literature on subjective well-being was extracted from Western social-cultural backgrounds, more studies are awaited in Eastern societies. 

In the Philippines, research on the mental health of senior citizens appears to be limited. Recently, mental disorder among Filipinos has been increasing, affecting around 17–20% of adults and 10–15% of children [30]. So far, we have examined the determinants of depressive symptoms among Filipino senior citizens in the Embracing and Nurturing Global Ageing (ENGAGE) research project [31]. In this population, psychological well-being might be a protective factor against depressive symptoms [31]. We also explored the unmet needs and coping mechanisms of Filipino senior citizens and identified their unmet needs, such as healthcare services, financial security, family support, and age-friendly environment [32]. Men and women cope differently to maintain their subjective well-being as they experience declining health and social support resources [32]. We hypothesized that there are gender differences in the factors associated with subjective well-being among Filipino senior citizens. Therefore, using the dataset of ENGAGE, this study aimed to examine the factors associated with subjective well-being between senior men and women living in a community in the Philippines. 

## 2. Methods 

### 2.1. Study Design and Setting 

We conducted a cross-sectional study as part of a situational analysis of the Embracing and Nurturing Global Ageing (ENGAGE) research project. The project ENGAGE was conducted in Muntinlupa City from 2017 to 2018. This action research project aimed to improve the psychological well-being of senior citizens living in the community. The research project was created in collaboration between the University of Tokyo and two institutions in the city: The Office for Senior Citizens Affairs (OSCA) and the City Health Office. The research project had three phases: situational analysis, peer counseling and leadership training, and an open, nonrandomized trial. Details about the training and trial have been reported elsewhere [33,34]. 

Muntinlupa City is located in the southernmost part of the National Capital Region (Metropolitan Manila). It is classified as a first-class, highly urbanized city. The city has a poverty incidence of 1.9% as of 2012 [35], and 5.6% of its total population (504,509) was comprised of senior citizens. Muntinlupa City is divided into two districts and had a total of nine barangays. In the Philippines, a “barangay” refers to a community or village with at least 2000 residents. The average household size in the city is 4.2 persons per household. 

### 2.2. Study Participants

In this study, participants were community-dwelling senior citizens in Muntinlupa City. From October to December 2017, we conducted a face-to-face survey interview among senior citizens. Participants had to be 60 years old and above and have a valid senior citizen identification (ID) card to be eligible in the study. The senior ID refers to the card issued by the OSCA office of the municipality or city where the participant lives. This locally issued ID is honored nationwide and serves as a proof of being a senior citizen [36]. We excluded in the study senior citizens who live in the nursing homes, with moderate/severe dementia, or with severe/life-threatening illnesses. We also excluded senior citizens who have problems in communication and suffering from impaired hearing. This study targeted only senior citizens living in the community and capable of answering the survey interview. 

### 2.3. Data Collection

We held a two-day training for data collection. Fifteen barangay health workers (BHWs) participated in the training. We explained the recruitment and data collection procedures, which include informed consent communication and ethical considerations. We also emphasized the importance of consistent interview methods. In all, two experienced researchers and 15 trained BHWs conducted the data collection using a structured questionnaire. 

We could not obtain the complete list of senior citizens living in Muntinlupa City. In this case, we used the list of senior citizens available in the barangay. The trained BHWs and experienced researchers recruited the senior citizens purposively by visiting their houses. The purposive sampling took into account the percentage of senior citizens per barangay. All senior citizens who were approached through home visits met the inclusion criteria and participated in the survey. Overall, we recruited 1021 senior citizens, and the duration of each survey interview was about 30 min. 

### 2.4. Variables and Measurements

We described the instruments used in this study based on previous research [31,37]. For the translation and adaptation of instruments, we followed the guideline from the World Health Organization (WHO) [38]. 

#### 2.4.1. Outcome: Subjective Well-Being 

The 5-item WHO well-being index is a short and generic global rating scale that measures subjective well-being. We used the WHO-5 to measure senior citizens’ subjective well-being. The scale reflected both the experiential (hedonic) and evaluative (eudaimonic) aspects of subjective well-being [39]. Senior citizens were asked to rate how well each of the five positive statements applied to them within the last 14 days. The five statements included having felt cheerful and in good spirits, active and vigorous, calm and relaxed, daily life filled with interesting things, and woke up feeling fresh and rested. Each of the five items was scored from not present (0) to always present (5). The total WHO-5 score was the sum of the five items, and the higher the score, the higher was the level of subjective well-being. The scale has high clinimetric validity and high sensitivity and specificity [39]. It has been used as a generic scale for subjective well-being and a screening tool for clinical depression worldwide [39]. The Cronbach’s α of the WHO-5 for this study was 0.88.

#### 2.4.2. Exposure: Socio-Demographic and Health Characteristics 

We collected the socio-demographic and health characteristics of senior citizens that were likely to affect their subjective well-being based on previous research [15,31,37]. These characteristics included socio-demographics, such as age, marital status, education, pension, monthly income, and living arrangement. For the health characteristics, we included self-rated health, the presence of chronic diseases, drinking, and smoking habits. 

We treated age as a continuous variable. For marital status, we categorized senior citizens into married, never married, separated, and widowed. Educational attainment was grouped into no education, attended elementary school, high school, and college. Concerning pension, we asked whether they are receiving it or not. We also asked their monthly income and grouped them into good, average, poor, or no income. We assessed their living arrangement by asking them whether they lived with others or lived alone. We also asked them about the presence of chronic diseases and how they assess their general health status from very bad to very good. Concerning lifestyle, we classified their smoking habits as a non-smoker, ex-smoker, and current smoker. In contrast, drinking habits were grouped as non-drinker, occasional, or daily drinker.

#### 2.4.3. Exposure: Psychosocial Factors

##### Psychological Resilience

The Resilience Appraisal Scale (RAS) contains 12 items that measure the individual’s ability to cope with emotions, gain social support, and solve problems [40]. We used this scale to assess senior citizens’ psychological resilience. Senior citizens rated their responses on a five-point Likert scale from strongly disagree (1) to strongly agree (5). We obtained the total RAS-12 score by adding the raw scores. The possible total score ranged from 12 to 60. Senior citizens who obtained a higher score indicated a higher level of psychological resilience. For this study, the Cronbach’s α of the RAS-12 was 0.93.

##### Perceived Social Support

The Duke Social Support Index (DSSI) measures social support that an individual received from others. In this study, we used the 10-item DSSI to measure senior citizens’ perceived social support. DSSI-10 has two subscales: social satisfaction and social interaction [41]. The social interaction subscale asked about the number of social interactions the senior citizen had within the past week. The social satisfaction subscale, on the other hand, asked about the quality of those social interactions [41]. Hence, the DSSI-10 is the sum of these two subscales, with a possible score from 10 to 30. The higher the score, the higher the level of social support among senior citizens. The Cronbach’s α of the DSSI-10 for this study was 0.82.

##### Loneliness

In this study, we used the short-form UCLA Loneliness scale. It contains eight items (ULS-8) to assess senior citizens’ level of loneliness [42]. Senior citizens were asked to rate how each statement described their current situation. Each of the eight items is scored from never (1) to always (4), and the total ULS-8 score ranged from 8 to 32. Before summing up the scores, we reverse coded the response to the statements, “I can find companionship whenever I want” and “I am an outgoing person”. There is no cut-off score to define loneliness; however, the higher the ULS-8 score, the higher the level of loneliness. For this study, the Cronbach’s α of the ULS-8 was 0.82.

### 2.5. Data Analysis

We summarized the senior citizens’ socio-demographic and health characteristics using descriptive statistics and showed their distribution by cross-tabulation. Then, we calculated the overall scores of the scales used in this study (WHO-5, DSSI-10, RAS-12, and ULS-8). We stratified the analysis by gender to see gender differences in the factors affecting senior citizens’ subjective well-being. We used *t*-tests or one-way analysis of variance (ANOVA) for the bivariate analyses.

To identify the factors associated with subjective well-being, we performed multiple linear regression analysis. We included all the exposure variables in the regression model. Multicollinearity is not a concern in the model because we obtained variance inflation factor (VIF) values less than 2.0. It also met the multiple linear regression assumptions, including homoscedasticity, normal distribution of residuals, and the linear relationship between the outcome and exposure variables. After that, to ascertain the predictors of subjective well-being, we performed a hierarchical regression analysis. We created six models for this analysis. For Model 1, we adjusted for demographic variables, including the senior citizens’ age, sex, educational attainment, living alone, and marital status. Then, we added the economic variables (pension and monthly income) in Model 2. As for Model 3, we included health characteristics, such as chronic diseases, self-rated health, drinking, and smoking habits. To see the independent association of loneliness and perceived social support with subjective well-being, we created Model 4 (without social support) and Model 5 (without loneliness), respectively. Finally, we further adjusted Model 6 (full model) for all the psychosocial variables, including psychological resilience, perceived social support, and loneliness. All statistical analyses were conducted using Stata 13.1 (StataCorp, College Station, TX, USA), and the level of significance was set to 0.05 (two-tailed).

### 2.6. Ethical Considerations

This study was approved by the Graduate School of Medicine’s Research Ethics Committee, The University of Tokyo (SN 11641), and the University of the Philippines-Manila Research Ethics Board (UPMREB 2017-312-01). We ensured the respondents’ confidentiality and privacy as no personally identifiable information was included in the study. All senior citizens participated voluntarily and were free to withdraw from the study without harm or penalty. Before conducting face-to-face interviews, we secured written informed consent from the senior citizens and their legal guardians when necessary. We also obtained all required approvals and permits, such as a Memorandum of Understanding between the University of Tokyo and Muntinlupa City.

## 3. Results

### 3.1. Characteristics of Participants

Table 1 shows the general characteristics of the senior citizens. Of the 1021 senior citizens, 699 (68.5%) were women, and their mean age was 67.9 years (standard deviation (SD) 6.2). One-third of the senior citizens were men, and their mean age was 67.3 (SD 5.9). Regarding marital status, women were more likely to be widowed (43.6% versus 11.5%). Almost half of them (337, 48.2%) attended high school/college. Moreover, women were more likely to have no income (72.6% versus 61.5%) and had chronic diseases (84.8% versus 76.7%). For their living arrangement, the majority of senior citizens (92.1%) lived with others. Concerning their lifestyle habits, men were more likely to be ex/current smokers (59.9% versus 7.5%) and occasional/daily drinkers (47.5% versus 6.3%).

### 3.2. Factors Associated with Subjective Well-Being of Filipino Senior Citizens

Table 2 shows the factors associated with Filipino senior citizens’ subjective well-being stratified by gender. Among women, those who were separated (β = −0.07; 95% CI = −3.4, −0.7) and received pension (β = −0.07; 95% CI = −1.2, −0.0) were negatively associated with a higher level of subjective well-being; whereas, among men, those who had no education were negatively associated with a higher level of subjective well-being (β = −0.05; 95% CI = −3.9, −0.2), as compared with those who had a high school/college education. Both men and women who had “good/very good” self-rated health (men: β = 0.23; 95% CI = 1.3, 3.3; women: β = 0.17; 95% CI = 1.0, 2.4), had higher psychological resilience (men: β = 0.30; 95% CI = 0.1, 0.3; women: β = 0.26; 95% CI = 0.2, 0.3), and had higher perceived social support (men: β = 0.14; 95% CI = 0.0, 0.4; women: β = 0.19; 95% CI = 0.1, 0.4) was positively associated with a higher level of subjective well-being.

Table 3 shows the results of the hierarchical regression analysis. As shown in Models 4 and 5, loneliness (β = −0.11, *p* < 0.001) and perceived social support (β = 0.21, *p* < 0.001) were independently associated with subjective well-being. However, the association of loneliness with subjective well-being (β = −0.05, *p* = 0.070) became insignificant in Model 6 (the full model), which suggested that loneliness was greatly influenced by perceived social support. Model 6 explained a total of 29.7% of the variance of subjective well-being. Those who had a “good/very good” self-rated health were positively associated with a higher level of subjective well-being (β = 0.19, *p* < 0.001) compared with those with a “fair” self-rated health. In contrast, those who had “bad/very bad” self-rated health showed the opposite results (β = −0.09, *p* = 0.003). Among the psychosocial factors, psychological resilience had the strongest association with subjective well-being (β = 0.27, *p* < 0.001), followed by perceived social support (β = 0.19, *p* < 0.001). Loneliness did not show any statistically significant association with subjective well-being in the final model (β = −0.05, *p* = 0.070).

## 4. Discussion

In this study, self-rated health, psychological resilience, and perceived social support were associated with Filipino senior citizens’ subjective well-being. Psychological resilience was the most powerful predictor of their subjective well-being. Loneliness, however, was the only psychosocial factor not associated with subjective well-being.

As shown in the hierarchical regression analysis, the influence of socioeconomic factors (e.g., education, income, and pension) wanes after introducing the psychosocial factors in the final model. This result might imply that psychosocial, rather than socioeconomic factors, greatly influenced Filipino senior citizens’ subjective well-being. Of all the psychosocial factors, psychological resilience was the most powerful predictor of subjective well-being, followed by perceived social support. Of all the health-related variables, positive self-rated health was the strongest predictor of subjective well-being. In this study, both men and women who had higher psychological resilience and had “good/ very good” self-rated health showed a higher level of subjective well-being. According to a literature review, higher resilience is associated with a higher quality of life and better mental health among senior citizens [43]. A positive health perception also increased the life satisfaction among senior citizens in Germany and Spain [44,45]. Our findings demonstrate the importance of psychological resilience and positive health perception in improving Filipino senior citizens’ subjective well-being, where resources are limited compared with these European countries.

Another important finding was that loneliness was the only psychosocial factor not associated with subjective well-being. This means that loneliness may not affect subjective well-being. This finding must be interpreted with caution because the association of loneliness with subjective well-being was greatly influenced by perceived social support, as shown in this study. This result is new and warrants further investigation because there could be reverse causality between loneliness and perceived social support. There is also a lack of empirical evidence that includes prospective studies to discern the relationship between loneliness and perceived social support and subjective well-being. Despite the lack of evidence, the English Longitudinal Study of Aging reported that loneliness was not associated with the rate of change in hedonic well-being, but social isolation does [20]. However, we did not measure social isolation in this study.

Both men and women who had higher perceived social support were positively associated with a higher level of subjective well-being. This finding illustrates that a social network or social exchange was a significant predictor of subjective well-being, consistent with other studies [17,22,23,46,47]. For instance, among senior citizens in the United States, having socially meaningful relations were positively associated with their subjective well-being and quality of life [22,23]; also, the influence of the social network on subjective well-being was higher for women than men. Pinquart and Sörensen [17] have previously confirmed this finding in their meta-analysis, too.

However, women who were separated and received pension reported a lower level of subjective well-being. Separated women in this study might have lost a supportive and intimate relationship, which can help in dealing with life stress. According to the British Household Panel Survey, women seemed more adversely affected by multiple partnership transitions and take longer to recover from partnership splits than men [48]. This result highlighted the positive effect of marriage or living with a partner on life satisfaction and subjective well-being [24,25]. As for the pension, women might have put more value on social contacts than on financial resources, as previously stated. Financial assets were more strongly related to men’s subjective well-being than women [49,50,51].

As for men, those who were uneducated had a lower subjective well-being than those with a high school/college education. Being uneducated among men might result in considerable internal conflict regarding gender roles, which might harm their mental health. This finding is the same as Lai et al., where they reported that senior citizens in Hong Kong who had a lower level of education are more likely to suffer from low subjective well-being [52]. Previous studies reported that higher education was associated with better subjective well-being across numerous settings [53,54]. Among older Americans, higher education may lead to more positive psychological states, which in turn contribute to good health [55]. Hence, our findings indicate the importance of a higher level of education to improve the subjective well-being of male Filipino senior citizens.

This study has several limitations. First, due to the study’s cross-sectional nature, the factors only suggest but do not confirm a causal relationship. We could not rule out reverse causality between psychological resilience and subjective well-being. For instance, those who have a low subjective well-being may have low psychological resilience. Secondly, there might be other factors that were not covered in the study, such as frailty and physical activity, which might also affect senior citizens’ subjective well-being [56,57]. It will be interesting to include these factors in future studies. Thirdly, we used convenience sampling to recruit senior citizens. As we could not obtain the complete list of senior citizens in the city, our sampling procedure was based on senior citizens’ percentage per barangay. Fourthly, we conducted the study in one urban city, so we cannot generalize the results for all Filipino senior citizens. Data collection from other subgroups located in the province will provide more information. Finally, some of the instruments we used in this study (e.g., DSSI-10, RAS-12, and ULS-8) were adapted from previous research [37,58,59] and have not been validated in the Philippine context. To overcome this issue, we did forward and back translation meticulously, performed face-validity testing by asking the experts (gerontologist and psychologists), pretested the questionnaires, and confirmed their reliability. Notwithstanding these limitations, the findings of this study have strengths and implications for policymaking and future interventions. This study is the first step in highlighting the subjective well-being of Filipino senior citizens.

## 5. Conclusions

This study underscored the essential factors associated with subjective well-being among community-dwelling senior citizens in the Philippines. Psychological resilience, positive self-rated health, and perceived social support might be protective factors for low subjective well-being. To improve their subjective well-being, we should build psychological resilience and social support networks in the community. Therefore, the local government may conduct community-based resilience programs and promote active participation among senior citizens.

## Figures and Tables

**Table 1:** Characteristics of the community-dwelling Filipino senior citizens.

Characteristics	Total (*n* = 1021)		Men (*n* = 322)		Women (*n* = 699)		*p*-Value
	*n*	%	*n*	%	*n*	%	
Age, mean (SD)	67.7 (6.1)		67.3 (5.9)		67.9 (6.2)		0.154
Marital status							<0.001
Married	570	55.8	248	77.0	322	46.1	
Never married	81	7.9	28	8.7	53	7.6	
Separated	28	2.8	9	2.8	19	2.7	
Widowed	342	33.5	37	11.5	305	43.6	
Educational attainment							0.022
No education	15	1.5	4	1.2	11	1.6	
Elementary	481	47.1	130	40.4	351	50.2	
High School	422	41.3	148	46.0	274	39.2	
College	103	10.1	40	12.4	63	9.0	
Monthly income							0.001
No income	705	69.1	198	61.5	507	72.6	
Poor income	209	20.4	76	23.6	133	19.0	
Average income	100	9.8	46	14.3	54	7.7	
Good income	7	0.7	2	0.6	5	0.7	
Pension							0.268
Have	489	47.9	146	45.3	343	49.1	
Do not have	532	52.1	176	54.7	356	50.9	
Living arrangement							0.017
Alone	81	7.9	16	5.0	65	9.3	
Living with others	940	92.1	306	95.0	634	90.7	
Self-rated health							0.263
Very good	19	1.9	3	0.9	16	2.3	
Good	294	28.8	92	28.6	202	28.9	
Fair	504	49.3	152	47.2	352	50.4	
Bad	199	19.5	73	22.7	126	18.0	
Very bad	5	0.5	2	0.6	3	0.4	
Number of chronic diseases							0.002
None	181	17.7	75	23.3	106	15.2	
One	430	42.1	137	42.5	293	41.9	
Two or more	410	40.2	110	34.2	300	42.9	
Smoking habits							<0.001
Non-smoker	776	76.0	129	40.1	647	92.5	
Ex-smoker	179	17.5	140	43.5	39	5.6	
Current smoker	66	6.5	53	16.4	13	1.9	
Drinking habits							<0.001
Non-drinker	824	80.7	169	52.5	655	93.7	
Occasional drinker	192	18.8	148	46.0	44	6.3	
Daily drinker	5	0.5	5	1.5	0	0.0	
RAS-12 score, mean (SD)	46.3 (5.0)		45.7 (5.8)		46.6 (4.6)		0.008
DSSI-10 score, mean (SD)	22.5 (3.4)		22.3 (3.4)		22.6 (3.4)		0.130
ULS-8 score, mean (SD)	7.2 (3.8)		7.1 (3.9)		7.2 (3.8)		0.671

SD—standard deviation; RAS-12—12-item Resilience Appraisal Scale; DSSI-10—10-item Duke Social Support Index; ULS-8—8-item UCLA Loneliness Scale.

**Table 2:** Factors associated with subjective well-being of community-dwelling Filipino senior citizens stratified by gender.

Variables	Men (*n* = 322)			Women (*n* = 699)		
	β	*p*-Value	95% CI	β	*p*-Value	95% CI
Age	0.02	0.682	(−0.1, 0.1)	−0.01	0.813	(−0.1, 0.0)
Marital status (vs. Married)						
Never married	−0.02	0.687	(−2.1, 1.4)	−0.02	0.604	(−1.6, 0.9)
Separated	0.10	0.118	(−0.7, 6.3)	−0.07	0.003	(−3.4, −0.7)
Widowed	0.03	0.541	(−1.0, 1.8)	−0.05	0.151	(−1.1, 0.2)
Education (vs. High School/College)						
No education	−0.05	0.030	(−3.9, −0.2)	0.02	0.710	(−2.3, 3.4)
Elementary	−0.01	0.858	(−1.0, 0.9)	0.01	0.846	(−0.5, 0.7)
Monthly income (vs. No income)						
Poor income	−0.01	0.817	(−1.2, 1.0)	0.01	0.786	(−0.6, 0.8)
Average/Good income	0.03	0.557	(−0.9, 1.8)	0.05	0.138	(−0.2, 1.8)
Pension (vs. None)	−0.04	0.468	(−1.3, 0.6)	−0.07	0.047	(−1.2, −0.0)
Self-rated health (vs. Fair)						
Good/Very good	0.23	<0.001	(1.3, 3.3)	0.17	<0.001	(1.0, 2.4)
Bad/Very bad	−0.13	0.014	(−2.6, −0.3)	−0.05	0.155	(−1.4, 0.2)
Chronic diseases (vs. None)	0.00	0.937	(−1.0, 1.1)	−0.05	0.148	(−1.5, 0.2)
Living alone (vs. Living with others)	−0.03	0.602	(−2.7, 1.5)	0.01	0.724	(−0.9, 1.1)
Smoking (vs. Non-smoker)	0.05	0.300	(−0.4, 1.3)	−0.05	0.157	(−2.1, 0.3)
Drinking alcohol (vs. Non-drinker)	0.00	0.948	(−0.9, 0.9)	0.02	0.475	(−0.8, 1.6)
Psychological resilience	0.30	<0.001	(0.1, 0.3)	0.26	<0.001	(0.2, 0.3)
Perceived social support	0.14	0.041	(0.0, 0.4)	0.19	<0.001	(0.1, 0.4)
Loneliness	−0.08	0.150	(−0.2, 0.0)	−0.05	0.182	(−0.2, 0.0)

CI—confidence interval.

**Table 3:** Hierarchical regression analysis predicting the subjective well-being of community-dwelling Filipino senior citizens (*n* = 1021).

Variables	Model 1	Model 2	Model 3	Model 4	Model 5	Model 6
Age	−0.05	−0.02	−0.01	−0.00	0.01	0.01
Sex (vs. Female)						
Male	−0.01	−0.03	−0.01	0.02	0.01	0.01
Marital status (vs. Married)						
Never married	−0.02	−0.02	−0.03	−0.01	−0.02	−0.02
Separated	−0.01	−0.01	−0.02	−0.02	−0.02	−0.02
Widowed	−0.02	−0.02	−0.04	−0.03	−0.04	−0.04
Education (vs. High School/College)						
Elementary	−0.05	−0.05	−0.05	−0.01	0.00	0.00
No education	−0.08 *	−0.08 *	−0.07 *	−0.02	−0.01	−0.01
Living alone (vs. Living with others)	−0.00	0.00	0.01	0.00	0.01	0.01
Monthly income (vs. No income)						
Poor income		0.06	0.03	0.01	0.00	−0.00
Average/Good income		0.12 ***	0.08 **	0.04	0.04	0.04
Pension (vs. None)		−0.06	−0.06 *	−0.06 *	−0.05	−0.05
Self-rated health (vs. Fair)						
Good/Very good			0.23 ***	0.18 ***	0.18 ***	0.19 ***
Bad/Very bad			−0.14 ***	−0.10 **	−0.09 **	−0.09 **
Chronic diseases (vs. None)			−0.07 *	−0.04	−0.04	−0.04
Smoking (vs. Non-smoker)			−0.03	−0.01	−0.00	−0.00
Drinking (vs. Non-drinker)			0.01	0.00	0.02	0.02
RAS-12				0.34 ***	0.28 ***	0.27 ***
DSSI-10				−	0.21 ***	0.19 ***
ULS-8				−0.11 ***	−	−0.05
*R*^2^ (%)	1.3	3.1	13.6	27.5	29.5	29.7
*ΔR*^2^ (%)	1.3	1.8 ***	10.5 ***	13.9 ***	15.9 ***	16.2 ***

RAS-12—12-item Resilience Appraisal Scale; DSSI-10—10-item Duke Social Support Index; ULS-8—8-item UCLA Loneliness Scale; *R*^2^—variance; Δ*R*^2^—change in variance. Values are presented as standardized beta (β). Statistical significance indicated by * *p* < 0.05; ** *p* < 0.01; *** *p* < 0.001.
